# Understanding the Nano-bio Interfaces: Lipid-Coatings for Inorganic Nanoparticles as Promising Strategy for Biomedical Applications

**DOI:** 10.3389/fchem.2019.00343

**Published:** 2019-05-15

**Authors:** Alessandra Luchini, Giuseppe Vitiello

**Affiliations:** ^1^Niels Bohr Institute, University of Copenhagen, Copenhagen, Denmark; ^2^Department of Chemical, Materials and Production Engineering, University of Naples Federico II, Naples, Italy; ^3^CSGI, Center for Colloids and Surface Science, Sesto Fiorentino, Italy

**Keywords:** lipids, inorganic nanoparticles, lipid coating, biomembranes, theranostic

## Abstract

Inorganic nanoparticles (NPs) exhibit relevant physical properties for application in biomedicine and specifically for both the diagnosis and therapy (i.e. theranostic) of severe pathologies, such as cancer. The inorganic NP core is often not stable in aqueous suspension and can induce cytotoxic effects. For this reason, over the years, several coating strategies were suggested to improve the NP stability in aqueous solutions as well as the NP biocompatibility. Among the various components which can be used for NP coatings, lipids, and in particular phospholipids emerged as versatile molecular building blocks for the production of NP coatings suitable for biomedical application. The recent synthetic efforts in NP lipid coatings allows today to introduce on the NP surface a large variety of lipid molecules eventually in mixture with amphiphilic or hydrophobic drugs or active molecules for cell targeting. In this review, the most relevant examples of NP lipid-coatings are presented and grouped in two main categories: supported lipid bilayers (SLB) and hybrid lipid bilayers (HLB). The discussed scientific cases take into account the most commonly used inorganic NP for biomedical applications in cancer therapy and diagnosis.

**Graphical Abstract:**
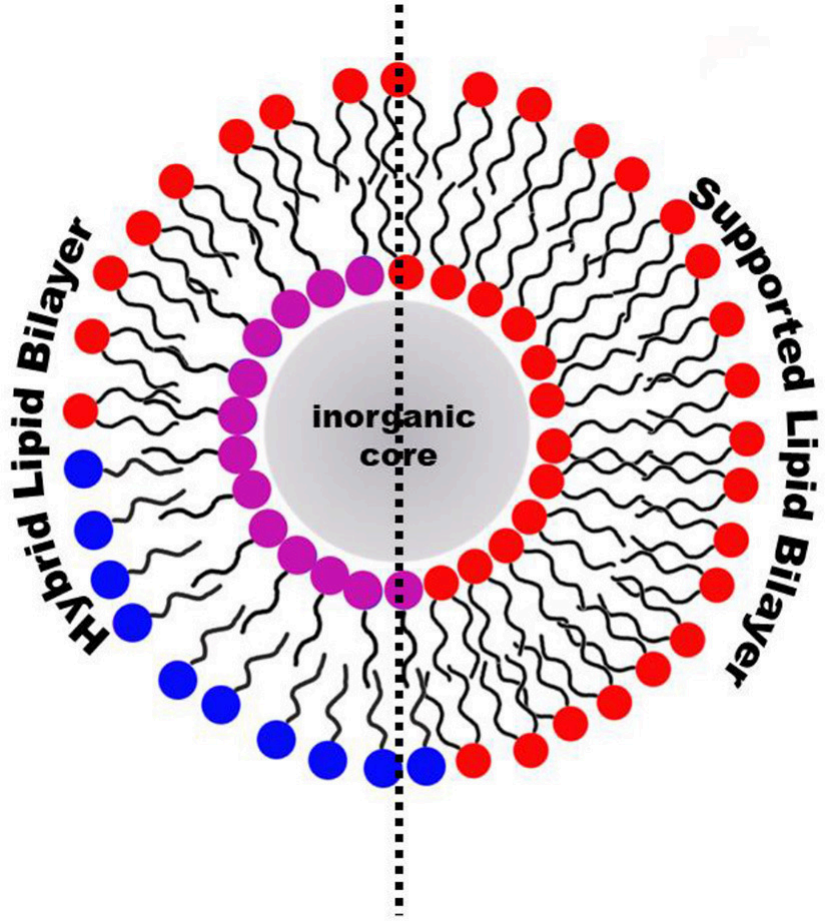
Hybrid Lipid Bilayer (HLB) and Supported Lipid Bilayer (SLB) are the most relevant lipid-coatings of inorganic nanoparticles for biomedical applications.

## Introduction

Nanomaterials of all sort of shapes and compositions have been designed in the last decades for biomedical applications and in particular in both the diagnosis and the therapy of cancer (Mahmoudi et al., 2011; Liang et al., 2014; Giner-Casares et al., 2016). The efforts in chemistry and physics to synthetize such systems and to understand their physico-chemical properties promoted the subsequent development of nanomaterials. More recently, the term “nano-biointerfaces” was introduced to highlight the convergence of nanotechnology and biomedicine, with the aim of implementing nanomaterials for nanodiagnosis, nanotherapy, and regenerative nanomedicine (Mahmoudi, 2018; Wang et al., 2018). Recent advances in nano-biointerfaces have further promised various functional biointegrated systems, including wearable and implantable nanobioelectronics (Dragoman and Dragoman, 2012; Cai et al., 2017) and nanomaterials for photochemical applications (Mahyad et al., 2015). Nevertheless, despite this rapid development, the bench-to-bedside translation of biomedical nanomaterials remains still challenging.

Lipids are natural constituents of cellular membranes where they provide the fundamental structural scaffold. Cell membranes are mainly composed by phospholipids and act as a barrier to protect the cell components from the surrounding environment (Simons, 2016). In analogy with their biological functions, lipids resulted to be good candidates for different biomedical applications where they provide a biocompatible protective barrier on the nanomaterial surface (Bothun, 2008; Weingart et al., 2013).

Liposomes or vesicles represented the first application of lipids in the developments of nano-biointerfaces (Soussan et al., 2009; Mo et al., 2014). Indeed, liposomes can be used to trap different kinds of molecules both hydrophilic in the inner aqueous compartment—or hydrophobic—in the lipid acyl chain region. Within the liposomes, the lipids act as protective barrier for the loaded molecular cargo and ensures its stability. The great advantage of liposomes is that the molecule loading is completely reversible and the loaded active molecules can be released once the liposomes reached the target organ or tissue (Huang et al., 2017). Liposomes have been successfully implemented for transporting and delivering drugs or probes for both therapeutic and imaging applications (Vitiello et al., 2015; Irace et al., 2017).

More recently lipids found another relevant application in the development of nanomaterials for biomedical applications (Namiki et al., 2011). Indeed, lipids are biocompatible molecules with an amphiphilic structure, which makes them the perfect candidates for inorganic nanoparticle (NP) surface coating.

Inorganic NPs present unique physical properties which can be exploited for imaging and therapy or simultaneous diagnosis and therapy, also known as theranostics (Caldorera-Moore et al., 2011) of cancer pathologies. Nowadays, several types of inorganic NPs have been suggested as suitable nanomaterials for the detection of tumors in their early stages as well as for their subsequent treatment (Yoon et al., 2017; Jiao et al., 2018). Nevertheless, their application is strongly limited by the high cytotoxicity which is often associated to them (Soenen et al., 2015). In this context, lipids and in particular phospholipids emerged as relevant candidates to produce nano-biointerfaces by coating the inorganic NP surface. NP coating with lipids improves the NP stability toward aggregation and reduces the related cytotoxic effects (Van Schooneveld et al., 2008; Nel et al., 2009).

This review specifically focuses on NP systems, where lipid molecules are used to coat the NP surface. The term “coating” is here used to generally refer to the introduction of organic molecules, such as lipids, on the NP surface. However, the coating processes are also referred to as NP functionalization, solubilization, or loading, which all indicate the introduction of a specific functionality in the NP system. As the main goal of this review is to report the current state of the art on the general use of lipids to coat the NP surface, we will not distinguish between the different types of coatings. Nevertheless, NP functionalization and solubilization can be used in the case of the lipid coating formation. The discussed scientific cases are limited to a selected type of inorganic NPs (silica, gold, silver iron oxide, and quantum dots), which distinguished among others for their large development in biomedicine (Li et al., 2012; Giner-Casares et al., 2016; Ni et al., 2017; Jiao et al., 2018). The role of lipids in providing NP coatings as well as the main strategies through which such coating can be achieved are here discussed and compared. Even though most of the reported scientific cases will involve the use of phospholipids, other kinds of lipids such as cholesterol or N-[1-(2,3-Dioleoyloxy)propyl]-N,N,N-trimethylammonium (DOTAP) will be presented as well.

Despite the rapid growth of the number of synthetic protocols for producing biomedical nanomaterials, their clinical translation is heavily compromised by the lack of a comprehensive understanding of biochemical interactions at nano-bio interfaces (Cai et al., 2018). The principal aspects of such interactions between lipid-coated NPs and biological interfaces will be discussed as a relevant aspect for a safe and effective application of biomedical nanomaterials.

## Lipids as Nanoparticle Coating Building Blocks

The coating of the NP surface emerged as a central step to guarantee NP colloidal stability in aqueous solution, enhanced biocompatibility as well as specific cell targeting functions (Bothun, 2008; Erathodiyil and Ying, 2011; Ladj et al., 2013).

The ability to combine different amphiphilic molecules with various functionalities in the NP coating corona brings further flexibility to the NP applications. Polymers, surfactants and lipids are largely used to provide the structural scaffold for organic NP coatings (Lin et al., 2008; Neoh and Kang, 2011; Pombo García et al., 2014; Campanella et al., 2015; Park et al., 2017).

**Figure 1:**
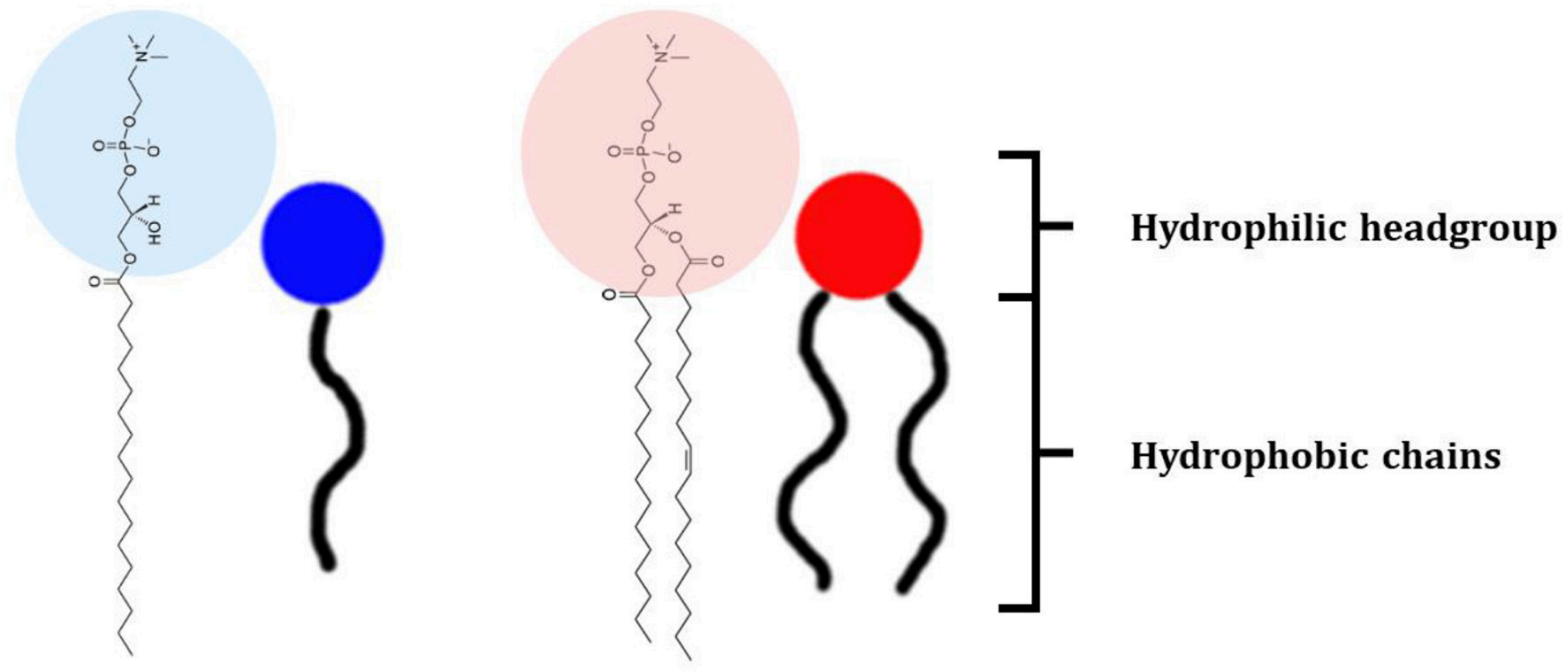
Schematic representation of the lipid structure. Lipid can exhibit a variable composition of both the headgroup and the acyl chains; in this review lipids are differentiated as baring one or two acyl chains and as function of the polar headgroup charge. As examples of commonly used mono-tailed (single acyl chain) and bi-tailed (two acyl chains) phospholipid, the molecular structure of 1-stearoyl-2-hydroxy-sn-glycero-3-phosphocholine (18LPC) and 1-palmitoyl-2-oleoyl-glycero-3-phosphocholine (POPC) are respectively reported. Additional lipid molecular structures are reported in Table 1.

The class of lipids identifies a large number of molecules with a wide variety of structures, but all characterized by a common structural motif [Figure 1; (Harayama and Riezman, 2018)]. Typically, the lipid molecule can be divided into a hydrophobic and a hydrophilic region. In particular, in phospholipids, the hydrophobic region is composed by one or two hydrocarbon chains whose length and level of unsaturation can be extremely variable (Table 1). The composition of the hydrophilic region can also be variable, so that lipid bearing negatively, positively, or zwitterionic groups are commonly found in nature (Coskun and Simons, 2011; Simons, 2016).

**Table 1:** Molecular structure of the lipids used in the discussed scientific cases.

**Zwitterionic phospholipids**	
1-palmitoyl-2-hydroxy-sn-glycero-3-phosphocholine (16LPC)	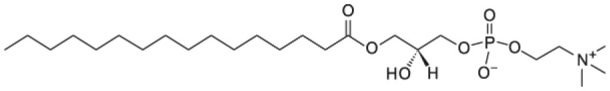
2-stearoyl-2-hydroxy-sn-glycero-3-phosphocholine (18LPC)	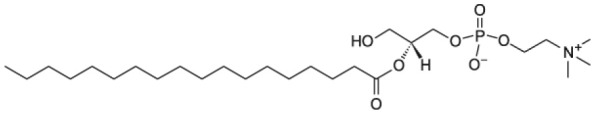
1,2-dimyristoyl-sn-glycero-3-phosphocholine (DMPC)	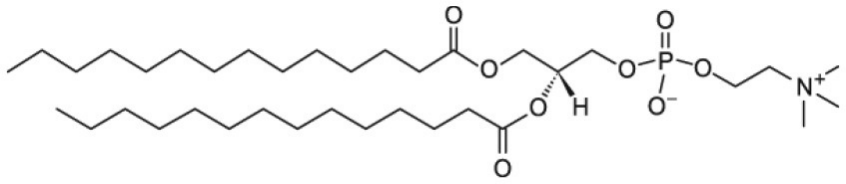
1,2-dipalmitoyl-sn-glycero-3-phosphocholine (DPPC)	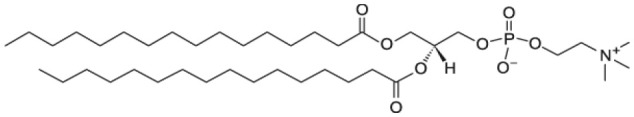
1-palmitoyl-2-oleoyl-glycero-3-phosphocholine (POPC)	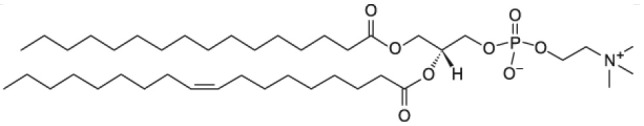
1,2-dioleoyl-sn-glycero-3-phosphocholine (DOPC)	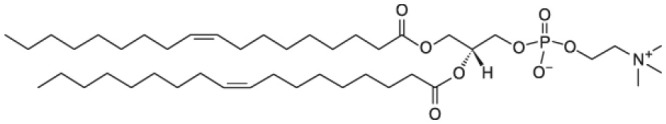
**Anionic phospholipids**	
1,2-dipalmitoyl-sn-glycero-3-phospho-(1′-rac-glycerol) (DPPG)	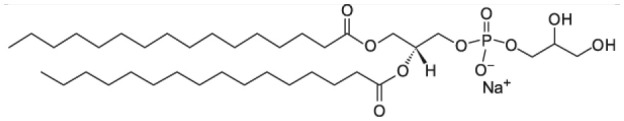
1-palmitoyl-2-oleoyl-sn-glycero-3-phospho-(1′-rac-glycerol) (POPG)	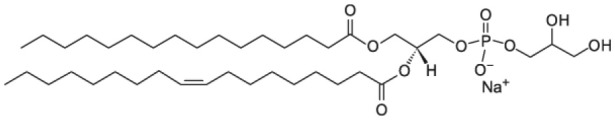
1-palmitoyl-2-oleoyl-sn-glycero-3-phospho-L-serine (POPS)	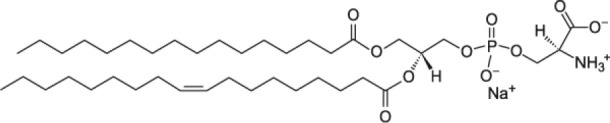
1,2-dioleoyl-sn-glycero-3-phospho-(1′-rac-glycerol) (DOPG)	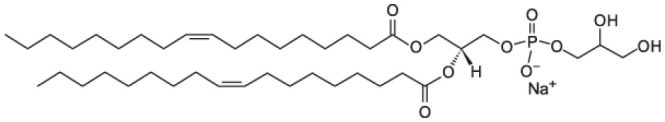
**Pegylated phospholipids**	
1,2-distearoyl-sn-glycero-3-phosphoethanolamine-N-[carboxy(polyethylene glycol)-2000] (PEG-DSPE)	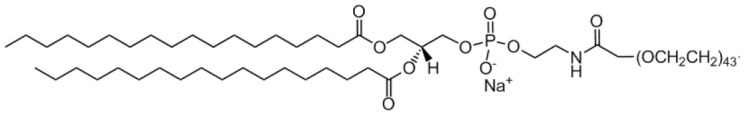
1,2-dipalmitoyl-sn-glycero-3-phosphoethanolamine-N-[azido(polyethylene glycol)-2000(PEG-DPPE)	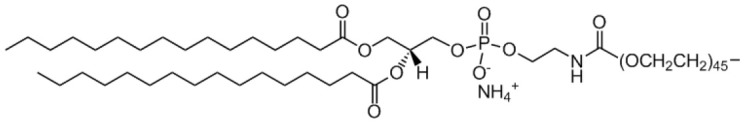
Cholesterol		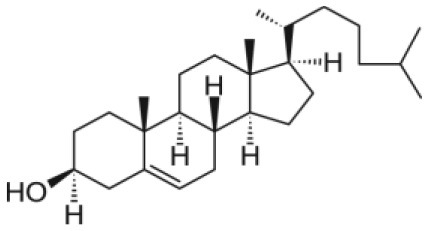
1,2-dioleoyl-3-trimethylammonium-propane (DOTAP)	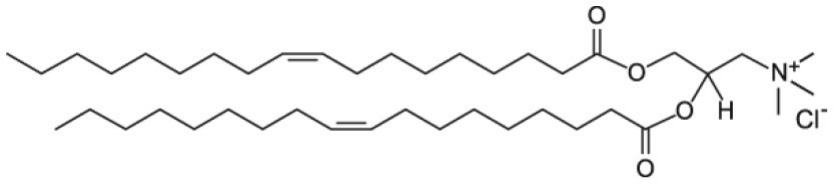

Among all, phospholipids (Figure 1) are the lipids with the molecular structure and amphiphilic properties that better matches the requirements for NP coating (Weingart et al., 2013). In particular, the zwitterionic phosphotidylcholine (PC) phospholipids are commonly used. NP colloidal stability can be improved by introducing charged molecules on the NP surface; charged molecules increase the inter NP repulsion, reducing the NP aggregation tendency. In this respect, mixtures of zwitterionic phospholipids and anionic or cationic phospholipids can be used for NP lipid coating. Another strategy to enhance the inter NP repulsion is to introduce on the NP surface molecules creating high steric hindrance when the NPs are close to each other, thus preventing aggregation. For this purpose, pegylated phospholipids combine the steric hindrance created by the long PEG chains with the biocompatibility of the hybrid lipid molecule (Suk et al., 2016).

As cellular membranes are characterized by negatively charged surface, it has been proposed that a positive surface charge can improve the uptake of biomedical nanodevices (Fröhlich, 2012). Hence, the cationic lipid DOTAP is as well a common choice for NP lipid coating.

Further details on the use of the above-mentioned lipids as well as other lipid systems are provided in the following sections.

## Strategies for Lipid Coating on Inorganic Nanoparticles

Lipid molecules can be introduced on the NP surface according to two different arrangements: supported lipid bilayers (SLB) and hybrid lipid bilayer (HLB). In the case of SLB, the lipids are adsorbed on the NP surface and adopt the typical arrangement of a lipid bilayer with the inner and the outer leaflet being composed by the same molecules. The lipid polar headgroups of the inner leaflet can favorably interact with the NP surface by means of both electrostatic and van der Waals interactions (Anderson et al., 2009). The main characteristic of the SLB coating is that the same lipid molecules are present both in the inner and outer leaflet. On the other hand, a HLB is formed when the inner and outer NP coating layer are not composed by the same type of molecules. Although the inner layer of the HLB, might still contain lipid molecules, i.e. fatty acids, its molecular composition is different from the outer leaflet, which can be composed by other lipid molecules. In the HLB, the two coating layers still adapt a lipid-bilayer-like arrangement, but the composition of the two leaflets is not identical and only the outer leaflet actually contains lipid molecules. For this reason, this type of coating is here named hybrid lipid bilayer. Because the SLB coating has the same composition of the inner and outer leaflet while the HLB coating has not, the protocols for producing the two types of coatings on the NP surface are substantially different. SLBs can be produced in a single step as the two leaflets are the same, while HLBs requires two separate steps to first produce the inner coating layer and subsequently to add the outer coating layer. A schematic representation of SLB or HLB coating on the NP surface is reported in Table 2 and further details on the two types of coating are reported in the following sections. Although the general concepts of the specific protocols for NP coating with either SLB or HLB are in principle transferable to other NPs, there are specific requirements that make a lipid-coating strategy mainly suitable for a selected number of NP systems.

**Table 2:** Schematic representation of the NP systems presented in the sections above.

**Coating strategy**	**NP core composition and diameter**	**NP coating composition**
Supported Lipid Bilayer 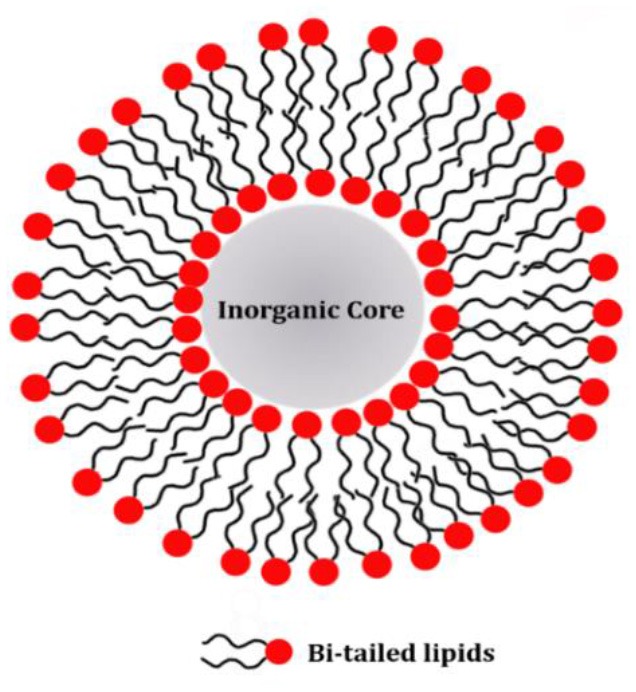	Silica (20–200 nm)	DOPC, DMPC, POPC, DOPS, DOTAP
	Gold (10–50 nm)	DOPG, DPPG, DPPC, PEG-DSPE, Cholesterol
	Silver (50–100 nm)	POPC, POPG
Hybrid Lipid bilayer 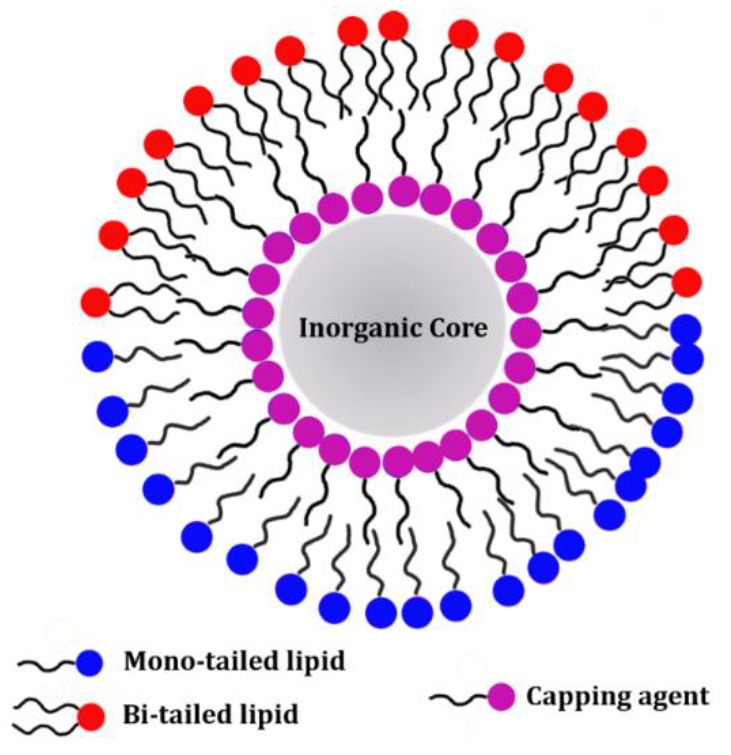	Gold (10–50 nm)	Inner layer: alkanethiol Outer layer: POPC, POPS
	Iron Oxide (5–50 nm)	Inner layer: oleic acid and oleylamine Outer layer: DOTAP, PEG-DSPE, 16LPC, 18LPC, DPPC, DPPG
	Quantum Dots (2–10 nm)	Inner layer: oleic acid and oleylamine Outer layer: PEG-DPPE

*The lipids listed in the table refers only to the scientific cases discussed in this review. Typical thicknesses of the NPs coating are 2–6 nm depending on the length of lipids used for the SLB or of the lipids and the capping agents used for the HLB*.

### Supported Lipid Bilayer

The formation of SLB or supported membranes on extended metal or metal oxide surfaces is a well-established phenomenon largely exploited for the production of artificial cell membranes, which are used in biostructural or functional studies and only recently also in technological applications (Richter and Brisson, 2005; Lind et al., 2014; Márquez and Vélez, 2017). In these cases, the lipid headgroups can stably interact with the surface of a hydrophilic substrate and the lipids arrange themselves as a two-layer system, where the first layer or leaflet is oriented with the headgroups toward the substrate and the acyl chains in the opposite direction. The second layer or leaflet is oriented in the opposite way, so that the acyl chains from both leaflets are facing each other and hence are protected from the aqueous solvent. In a very similar fashion, it has been suggested that lipid bilayers could be as well-formed on NP surfaces (Mornet et al., 2005). The main requirement that such a method has to meet is that the NPs exhibit a hydrophilic surface. Because the inner and outer leaflet of the SLB coating are formed during the same step of the NP coating, the two leaflet are composed by the same lipid molecules.

Silica NPs are very good candidates for SLB surface coating. In analogy with silicon substrates largely used for surface-sensitive techniques, a similar mechanism of SLB formation has been suggested for silica NPs (Mornet et al., 2005; Ahmed et al., 2011; Jing et al., 2014). Silica NPs are particularly suitable for this kind of lipid coating as they are intrinsically soluble in water or saline buffers and can meet the appropriate condition for vesicle fusion (Lind et al., 2014). Formation of SLB via vesicle-fusion has been widely reported as very efficient route to produce lipid-functionalized silica NPs, also known as protocells (Liu et al., 2009; Ashley et al., 2012; Dengler et al., 2013; Butler et al., 2016). Such coating strategy is based on a spontaneous process which occurs in aqueous solution by simply mixing the NPs with the lipid vesicles.

Several examples are available in the literature for SLB-coating of silica NPs, in which different kinds of phospholipids and other lipids were successfully implemented (Fu et al., 2015; Choi et al., 2016; Durfee et al., 2016). Particularly, bi-tailed phosphotidylcholines as 1,2-dioleoyl-sn-glycero-3-phosphocholine (DOPC), 1-palmitoyl-2-oleoyl-glycero-3-phosphocholine (POPC) or 1,2-miristoyl-sn-glycero-3-phosphocholine (DMPC) were used alone or in mixture with anionic phospholipid 1,2-dioleoyl-sn-glycero-3-phosphoserine (DOPS) or the cationic lipid DOTAP. As the silica NP surface is negatively charged, the use of positively charged lipids (e.g., DOTAP) facilitate the vesicle fusion process (Liu et al., 2009; Savarala et al., 2010).

**Figure 2:**
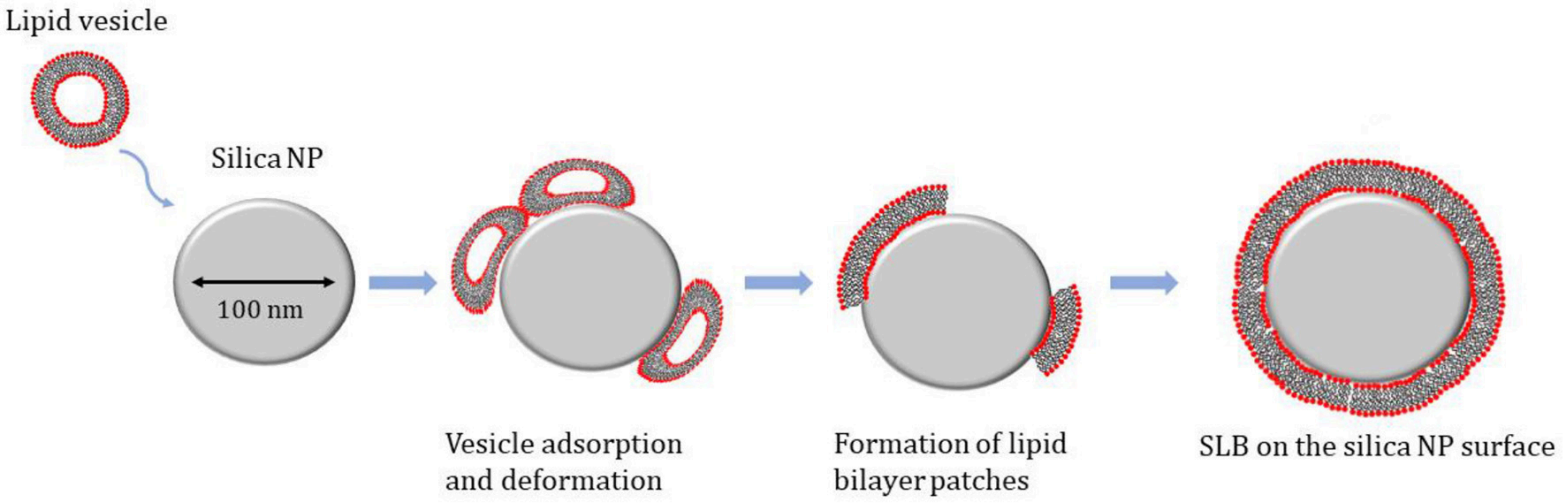
Schematic representation of the mechanism for the Supported Lipid Bilayer (SLB) formation on the surface of silica nanoparticles.

The specific mechanism for SLB formation on silica NPs can be schematically described as composed by 3 steps: (1) adsorption of vesicles on the NP surface; (2) deformation of the SUV and increase in the contact area; (3) rupture of the vesicles and formation of lipid patches, which eventually fuse and form continuous lipid bilayer on the NP surface (Figure 2).

Although less exploited compared to silica NPs, gold NPs (AuNPs) can also provide the appropriate surface for SLB formation (Li et al., 2008; Du et al., 2017; Mei et al., 2018). Two main strategies can be used to form a SLB on AuNP surface: (1) pre-synthesized gold seeds are grown to the final size in the presence of lipids (Bakshi et al., 2008); (2) pre-synthesized AuNP suspension is used to re-disperse a lipid film composed by phospholipids, such as the anionic phospholipid 1,2-dioleoyl-sn-glycero-3-phospho-(1'-rac-glycerol) (DOPG) (Bakshi et al., 2007). In the first approach, the lipids coat the NP surface during the inorganic core formation and limit the growth of the AuNPs to a specific size. On the other hand, in the second approach, the AuNPs synthesis and the formation of the SLB are two separate steps. This latter strategy of forming SLB on AuNP surface is particularly flexible in terms of the lipid species that can be introduced on the NP surface, as an example it allows the use of phospholipids such as 1,2-dipalmitoyl-sn-glycero-3-phosphocholine (DPPC) and 1,2-dipalmitoyl-sn-glycero-3-phospho-(1′-rac-glycerol) (DPPG) as well as cholesterol and pegylated lipids, e.g., 1,2-distearoyl-sn-glycero-3-phosphoethanolamine-N-[amino(polyethylene glycol)-2000] (PEG-DSPE) (Kang and Ko, 2015). Both the above-mentioned protocols lead to a similar final structure.

A relevant test to verify the effective coating of the AuNPs by the lipids is the oxidation with NaCN of the uncoated AuNPs. In this test the lipid membrane formed on the NP surface acts as a non-ion-permeable barrier protecting the inorganic core from being exposed to NaCN (Hamilton et al., 2017). This test also highlights that the SLB actually screens the inorganic core from being exposed to the surrounding media without affecting its optical properties which are relevant for the biomedical application. This is an important aspect as it has been previously reported that the NP surface might act as suitable platform for protein and other biomolecule aggregation (Monopoli et al., 2011) with consequent cytotoxic effects.

As AuNPs, also the silver NP (AgNP) surface can be used as platform for SLB formation. Typically, AgNPs are initially synthesized in an organic solvent, which is subsequently evaporated and a film of AgNPs is formed. The dried NPs are re-dispersed with an aqueous solution containing a mixture of the zwitterionic phospholipid POPC and the anionic phospholipid 1-palmitoyl-2-oleoyl-sn-glycero-3-phospho-(1′-rac-glycerol) POPG (Bhowmik et al., 2015). Although this approach effectively produces AgNPs with a lipid coating, it might lead to the formation of NP aggregates. During the re-dispersion step, more than one inorganic core can be coated by the SLB; sonication of the AgNPs suspension can reduce the size of the NP aggregates.

### Hybrid Lipid Bilayer

Frequently, NP synthesis is performed in the presence of capping agents—small molecules or surfactant which can on the NP surface during NP synthesis (Rao et al., 2012; Wu et al., 2016). The main role of the capping agents is to control NP growth during the synthesis and to prevent aggregation and precipitation (Hao et al., 2010). The capping agent layer can be either kept during a subsequent NP coating step or it can be replaced by another layer of molecules during the so-called ligand-exchange process (Woehrle et al., 2005). Typically, the capping agent molecules exhibit a hydrocarbon chain of variable length which will be exposed toward the solvent once the NP surface is coated (Lu et al., 2007). For this reason, the NPs will be mainly soluble in non-polar solvents. However, the capping agent chains can be used as a platform to further coat the NP surface with a second layer of amphiphilic molecules. The result is the formation of a HLB, as the two leaflets do not show the same molecular composition (also referred to lipid micelle; Mulder et al., 2009).

Amine, carboxylic acids and thiols are among the most used capping agents during metal or metal oxide NPs or quantum dots (QDs) synthesis (Rao et al., 2012). The amino, carboxyl or thiol functional group can bind the metal atoms at the NP surface and form stable bonds between the NP and the coating molecules. Although several aspects of such bonds are still to be fully characterized, they are commonly referred to as covalent bonds (Laurent et al., 2008; Rao et al., 2012). Some of the above mentioned capping agents also belong to the class of lipids as in the case of the oleic acid. Nevertheless, the main feature distinguishing the HLB from the SLB is that different molecules (and in some cases different lipids) are composing the inner and outer leaflet.

**Figure 3:**
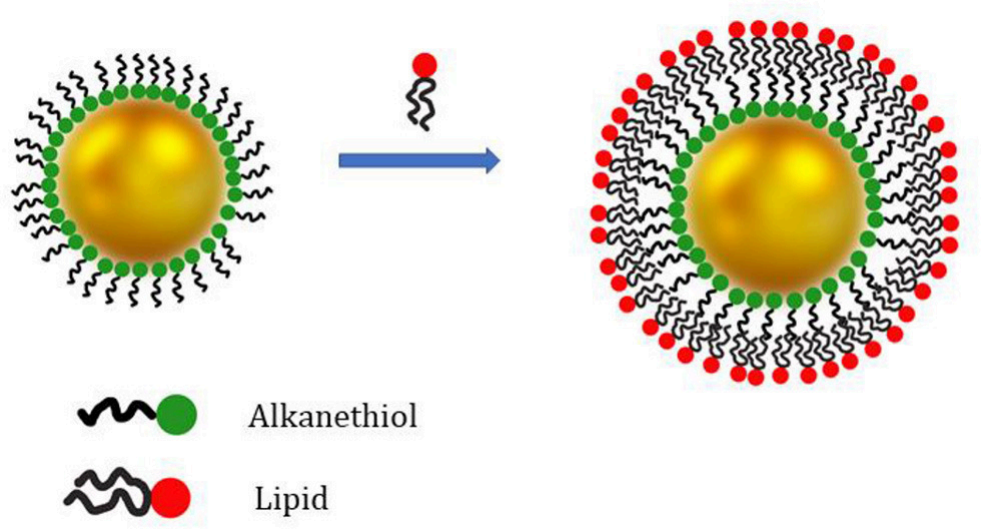
Schematic representation of AuNPs coating with a hybrid lipid bilayer. The inner coating layer is composed by alkanethiol molecules. The AuNPs exhibiting only the alkanethiol coating layer are only soluble in non-polar solvents. Addition of phospholipids or other lipids (e.g., DOTAP) leads to the formation of a second coating layer on the AuNP surface (formation of the HLB). The outer lipid layer ensures AuNP stability in aqueous suspension.

The formation of a HLB is a suitable strategy to introduce lipids on the surface of metal and metal oxide nanoparticles. Among all, thiols are well known to stably bind gold surfaces and thus they are frequently used for AuNPs (Woehrle et al., 2005). Indeed, the SLB coating preparation protocols often involve a large amount of unbound lipids which might be difficult to be completely removed from the solution and can affect the results of biological membrane binding studies. In this respect, Alkanethiols are used to improve the overall stability of the lipid coating and facilitate the removal of the lipid excess (Hamilton, 2017). As an example, the sequential addition of POPC:POPS (70:30) liposomes and propanethiol to freshly synthesized AuNP successfully produces a HLB coating on the NP surface in a single step. The thiol groups of the propanethiol molecules form a first coating layer on the AuNP surface. On the other hand, POPC and 1,2-dioleoyl-*sn*-glycero-3-phosphoserine (POPS) phospholipids self-assemble on the AuNPs surface coated with the propanethiol molecules and form a second amphiphilic layer (Figure 3). The liposome$\bar{s}$ excess is removed *via* centrifugation (Hamilton et al., 2017).

A very convenient strategy to produce a lipid coating on iron oxide-based NPs is to produce a HLB with the inner leaflet composed by commonly used capping agent molecules, i.e., oleylamine and oleic acid (Laurent et al., 2008). As an example, oleic acid and oleylamine protected superparamagnetic iron oxide nanoparticles (SPIONs) are mixed in chloroform with DOTAP and a pegylated phospholipid, (PEG-DSPE). The organic solvent is evaporated thus producing a film including both the lipids and the SPIONs. The film is subsequently re-hydrated with water and intensively sonicated to reduce NP aggregation (Huang et al., 2009). As a result, a HLB coating with the oleic acid and oleyamine molecules in the inner leaflet and the DOTAP and PEG-DSPE is produced.

**Figure 4:**
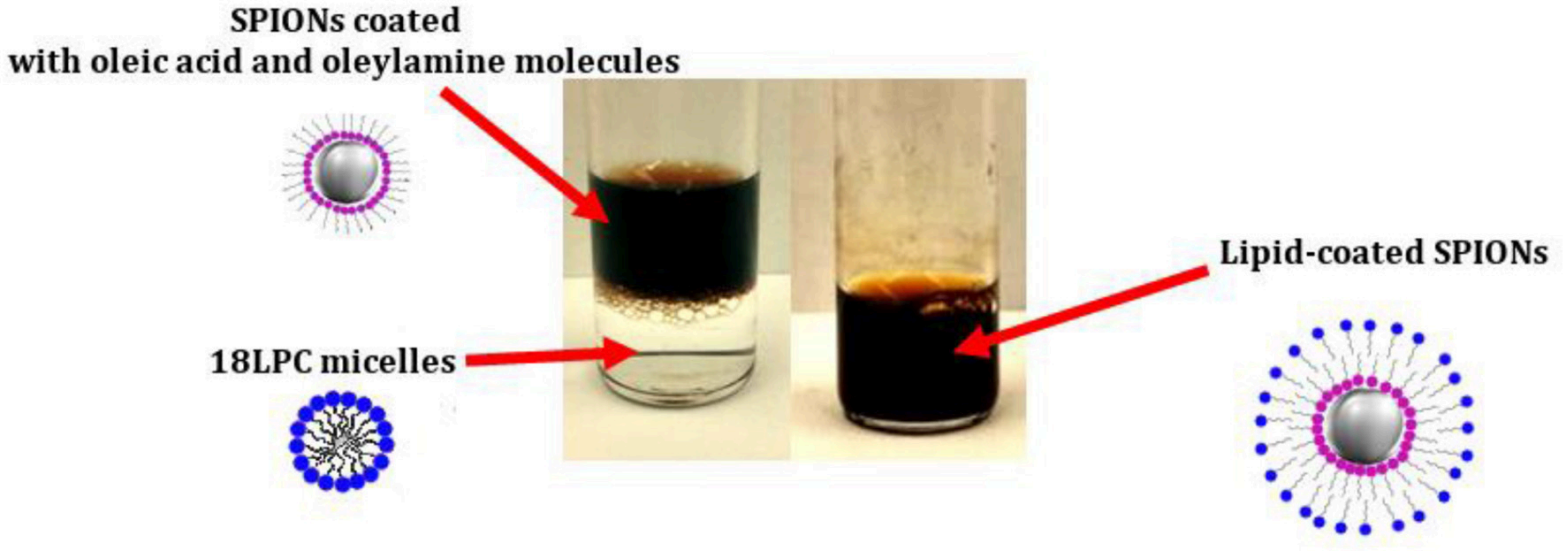
Schematic representation of the SPION coating with 18LPC molecules [adapted from (Luchini et al., 2016b), reproduced by permission of The Royal Society of Chemistry]. The biphasic system with the transparent 18LPC solution in the bottom and the dark-colored SPION suspension is reported on the **(Left)**. Upon sonication and evaporation of the organic solvent a single phase system containing the 18LPC-coated SPIONs in water is formed (picture on the **Right**).

Lysophosphatidycholine (LPC) molecules can also be used to stabilize SPIONs in aqueous solution. LPC is a class of phospholipids characterized by a single acyl chain and thus exhibiting a surfactant-like behavior in solution (Figure 1). Indeed, while bi-tailed phospholipids such as POPC or DOPC, mainly form liposome in solution, LPC can form micelles (Vitiello et al., 2009). The dynamic nature of LPC micelles in solution makes the LPC molecules suitable candidates for NP coating (Luchini et al., 2016a). SPIONs can be coated with LPC molecules via a solvent-transfer approach (Luchini et al., 2016b). In particular, the cyclohexane dispersion of SPIONs coated with oleic acid and oleylamine molecules is added to the aqueous solution containing the LPC molecules. The so-formed bi-phasic system is sonicated at 50°C to fully evaporate the organic solvent and promote the transfer of the SPIONs into the water phase where they are stabilized by the LPC coating (Figure 4). Hence, a HLB composed by the oleic acid and oleylamine inner leaflet and the LPC outer leaflet is formed on the SPION surface. This method was successfully tested for different kinds of LPCs (different acyl chain length) as well as surfactants. The formation during the coating process of SPION aggregates covered with LPC molecules is strongly correlated to the cmc of the used LPC or surfactant. Thus, the LPC with the lowest cmc, i.e., 18LPC, resulted the best choice for producing single SPIONs coated with the HLB and avoiding SPION aggregation (Luchini et al., 2016a). NP coating with LPC molecules through the solvent-transfer approach was as well-extended to AuNPs exhibiting an oleylamine capping layer (Luchini et al., 2018).

HLB coatings can be produced on the QD surface such as CdSe/CdS/ZnS core-shell NPs (Michalet et al., 2005). As SPIONs, QDs are often synthesized with a hydrophobic capping agent layer (Zhou et al., 2015). As an example, QDs can be synthesized with a hydrophobic coating composed by octadecylamine and oleic acid. This hydrophobic layer on the NP surface can be subsequently coated with lipids or PEGylated lipid molecules, 1,2-dipalmitoyl-sn-glycero-3-phosphoethanolamine-N-[azido(polyethylene glycol)-2000 (PEG-DPPE) (Zhao et al., 2014). In particular, the pre-synthesized QDs with the capping agent coating and the lipids are both dispersed in chloroform and dried to form a film containing both the NPs and the lipids. The film is subsequently dispersed in water or buffer. QD aggregate formation might occur during the lipid outer leaflet formation; however ultracentrifugation of the aqueous suspension can be used to remove the QD aggregates and obtain a well-dispersed NP suspension (Mohs et al., 2009).

## Interactions of Lipid-Coated Nanoparticles With Biomembranes

The interaction of NPs with the biological environment, i.e., cells and lipid biomembranes, modulates the NP internalization in cells and determines the efficacy of their use in biomedicine, from phototherapy to the imaging, from drug/gene delivery to theranostics (Verma and Stellacci, 2010). Indeed, these applications require a steady control over NP–biosystem interactions, which are mainly influenced by the NP features, such as size, morphology and surface properties (Ramishetti and Huang, 2012; Hoshyar et al., 2016). Consequently, understanding the interactions between uncoated or coated-NPs and biological systems is of high-priority interest to elucidate the physical mechanisms of NP potential toxicity (Nel et al., 2009).

In this context, the interactions of lipid-coated NPs with cells and bio-mimicking membranes has not been widely studied yet, whilst seemingly essential to evaluate the NPs effectiveness as potential drug delivery vehicles and/or bioimaging labels. Although an increasing attention is actually focused on this field, many other features could be deepened to shed light on the driving forces modulating the interactions of lipid-coated NPs and lipid biomembranes. In this section, we try to elucidate the state of the art on the interaction between lipid-coated NPs and cell membranes or model bio-membranes.

### NP-Cell Interaction

The plasma membrane is a selectively permeable barrier whose biological function is to protect and maintain the essential intracellular environment of the cell. The binding, uptake and safe localization of the NPs into the cytosol may be critical in biomedical applications (Nel et al., 2009; Lin et al., 2010). However, crossing cell membranes is inherently challenging due to the nature of the lipid bilayers and as well as to the NP physico-chemical features.

Lipid-coated silica NPs are among the most studied (Lee et al., 2011; Li et al., 2012). Experimental evidences demonstrated that the lipid-coating, composed by a SLB of DOPC and DOPG at equimolar mixture, did not cause significant changes in the photophysical properties of silica NPs containing methylene blue as photosensitizer for the biomedical application (Tada et al., 2007, 2014). However, bare and lipid-coated NPs exhibited a completely different interaction with cells. The lipid-coated silica-NPs were distributed through the cell cytoplasm whereas the bare NPs were detected only in some vacuolar regions within the cells (Tada et al., 2014). On the other hand, lipid-coated silica NPs loaded with four drugs (irinotecan, 5-fluorouracil, oxaliplatin, and leucovorin) against the pancreatic ductal adenocarcinoma were recently designed and tested on cells. The lipid coating, composed by a mixture of 1,2-Distearoyl-sn-glycero-3-phosphocholine (DSPC), cholesterol and 1,2-distearoyl-sn-glycero-3-phosphoethanolamine-N-[methoxy(polyethyleneglycol)-2000] (DSPE-PEG2000), determined a strong reduction of the drug toxicity, an increase in NP stability toward aggregation, as well as a more favorable NP internalization with respect to liposomes (Liu et al., 2016). Similar results were also obtained for silica NPs, coated with a SLB formed by DPPC/cholesterol/1,2-distearoyl-sn-glycero-3-phosphoethanolamine (DSPE)-PEG, which were favorably tested as nanocarriers able to synergistically co-deliver gemcitabine and paclitaxel drugs against the human pancreatic cancer in mice (Meng et al., 2015). A high specificity and efficiency were also observed for peptide-targeted lipid-coated silica NPs, also known as “protocells,” which exhibited multiple properties overcoming many of the limitations of the existing delivery platforms. The multifunctional lipid-coating, composed by a SLB of DOPC/DOPE/cholesterol/1,2-dioleoylsn-glycero-3-phosphoethanolamine-N-[methoxy(polyethylene-glycol)-2000] (18:1 PEG_2000_PE) mixtures, promoted the NP endosomal escape and cytosolic dispersion (Ashley et al., 2012).

Altogether the above-mentioned scientific cases demonstrate that the lipid coating strongly improved crossing the cell membranes and entering into the cells by silica NPs.

Interestingly, AuNPs coated with a SLB composed by 1-stearoyl-2-oleoyl-*sn*-glycero-3-phospho-(1′-*rac*-glycerol) (SOPG) have been observed to localize in acidic compartments of A549 cells, inducing the formation of lamellar bodies (LBs) (Wang and Petersen, 2013). Also, AuNPs coated with a SLB formed by dimethyldioctadecylammonium bromide (DODAB)/dioleoyl-phosphatidylethanolamine (DOPE) were synthesized to obtain a new DNA-based vector. The presence of the DOPE phospholipid favored the interaction of AuNPs/DNA complexes with cell membranes, increasing their internalization and promoting the DNA release from early endosomes, due to a major sensitivity to the anionic lipid membrane and to the decreasing pH along the endocytic pathway (Du et al., 2015).

### NP-Biomimicking Membrane Interaction

Cell membranes are very complex systems as they include a large variety of different lipid and protein species. Hence characterizing the interaction of NPs and cell membranes requires the use of model systems which are able to reproduce the fundamental properties of cell membranes, but at the same time exhibit a reduced molecular complexity. Liposomes and supported membranes on solid substrates are among the most important lipid-based systems for mimicking cell membranes (Tien and Ottova-Leitmannova, 2000), which are commonly used to investigate the effect of NPs on cell membrane biomimics (Tatur et al., 2013; Heikkilä et al., 2014; Simonelli et al., 2015; Troiano et al., 2015; Malekkhaiat Häffner and Malmsten, 2017). However, fewer studies are actually reported in the literature on the interactions between lipid-coated NPs and lipid membranes. The cell membranes are among the most relevant biointerfaces that NPs will encounter upon administration, and the interaction with such interfaces has a pivotal role during NP uptake by the targeted cells. Clearly, a major attention on this field appears necessary to clarify the driving forces influencing NP-biological membrane interaction.

In this context, the effect of bare silica NPs and silica NPs coated with a DOPC/DOPG SLB on supported membranes composed by DOPC/DOPG phospholipids was recently studied. The supported membranes were prepared at two different molar ratios (4/1 and 1/4), in order to specifically discriminate the role of negatively charged and neutral lipids, respectively, in modulating the biomembranes-NPs interaction (Tada et al., 2007). Experimental evidences, obtained by Surface Plasmon Resonance (SPR) Imaging, demonstrated that the bare silica-NPs exhibited a high adsorption and aggregation on the supported membranes, independently of the membrane surface charge. On the other hand, the lipid-coated NPs exhibited less aggregation tendency on the membrane surface and the adsorption was dependent on the charge of both the NPs and the supported membranes. These results clearly confirmed that the decisive role of DOPC/DOPG lipid coating in modulating the NPs aggregation process on the membrane surface, consequently affecting their cytosolic distribution (Tada et al., 2007).

**Figure 5:**
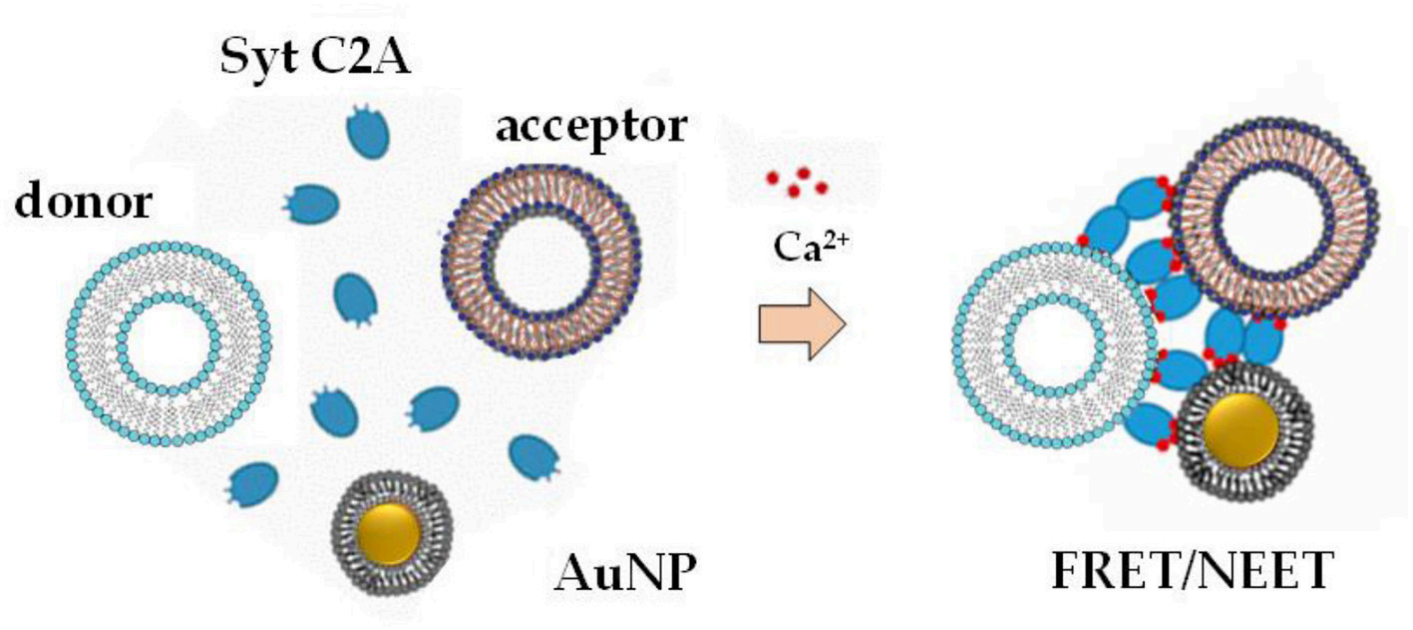
Mechanism of liposomes clustering mediated by lipid-coated AuNPs [adapted with permission from (Hamilton et al., 2017). Copyright (2017) American Chemical Society].

Lipid-coated AuNPs were used as biosensors, due to their high extinction coefficients and a relatively inert behavior, to monitor liposomes clustering mediated by specific domains of synaptotagmin proteins in the presence of Ca^2+^ ions (Hamilton et al., 2017), using a combined approach of Förster Resonance Energy Transfer (FRET) assays, dynamic light scattering (DLS) and spectroscopic techniques (Figure 5).

Recently, the interactions between AuNPs coated with a HLB of neutral and/or charged ligands and phase-separated lipid bilayers of DPPC/C18:3-dioctadecatrienoyl-phosphocholine (DFPC)/cholesterol (4:3:3 molar ratio) was investigated by using coarse-grained Molecular Dynamics (MD) (Chen et al., 2018), focusing on both the penetration and the adsorption processes as well as the final distribution of lipid-coated AuNPs ligand density and the surface charge of the NPs which strongly drive the penetration into the liquid-disordered (L_d_) or liquid-ordered (L_o_) region of the biomimicking lipid bilayer. These insights could appear very useful to better define the lipids chemical features to improve the efficiency of designing lipid-coated nanoparticles for specific applications (Chen et al., 2018).

**Figure 6:**
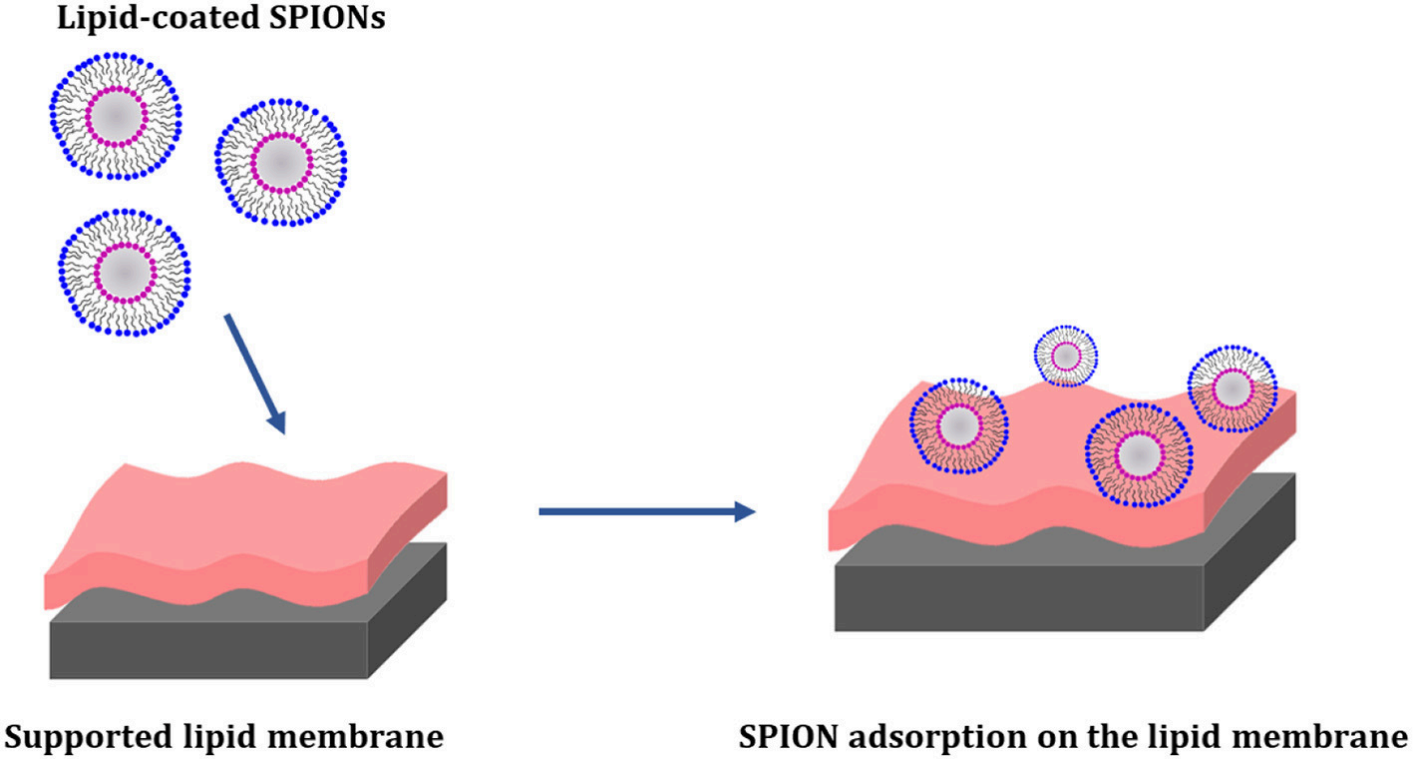
Schematic representation of the interaction between 18LPC/SPIONs and supported membranes composed by POPC/POPG/cholesterol. Neutron reflectometry confirmed the absorption of the nanoparticles on the membrane surface. The 18LPC/SPIONs were found to interact only with the phospholipid headgroups (Luchini et al., 2017).

The interaction between lipid coated SPIONs and supported membranes composed of the zwitterionic phospholipid POPC, the anionic phospholipid POPG and cholesterol, as mimics of the mammalian plasma membrane, was recently investigated by Neutron Reflectometry (NR) and Quartz Crystal Microbalance with Dissipation monitoring (QCM-D) (Luchini et al., 2017). Two different HLB-coated SPIONs, alternatively with a cationic surfactant (CTAB) and a zwitterionic (18LPC) phospholipid, were prepared and put in contact with the supported membranes containing different amounts of cholesterol. Experimental results indicated that the lipid-coated SPIONs adhere to the surface of the lipid membranes without removing lipids from the membrane and that the attachment is strongly dependent by the cholesterol content. The 18LPC-coated SPIONs were found to adsorb on the bilayer surface resulting in a highly hydrated layer with low density of SPIONs. This interaction between the lipid-coated SPIONs and the lipid bilayers do not affect the membrane overall structure (Figure 6).

## Lipid-Coated Nanoparticles Application in Nanomedicine

The NP inorganic core can provide drug-carrier (silica NPs), diagnostic (gold, iron oxide NPs, and QDs), therapeutic (gold, iron oxide NPs), and biosensor (silver NPs) functions, depending on the chemical nature of the inorganic component (Mirahadi et al., 2018). Among the desirable characteristics of an ideal NP-based biomaterial are: (1) carrying high levels of multiple diverse molecular cargos; (2) long circulation time in blood; (3) selective binding of target cells; (4) controlled release of the molecular cargo.

Many of the above-mentioned aspects can be addressed by silica NPs exhibiting a lipid coating. Indeed, silica NPs can encapsulate active molecules such as drugs or diagnostic agents or enzymes (Pavel et al., 2018) in the mesoporous nanoparticle core, preventing their potential degradation (Ashley et al., 2012). On the other hand, the NP lipid coating improves NP biocompatibility and eventually controls the release of the active molecules from the NP core. Lipid digestion and the NP core overtime degradation in the non-toxic silica acid and related by-products make lipid-coated silica NPs highly biocompatible (Pavel et al., 2018). The biocompatibility of silica nanoparticles, coated with a SLB of 1,2-distearoyl-*sn*-glycero-3-phosphoethanolamine-*N*-[methoxy(poly(ethylene glycol))-2000] (PEG_2000_-DSPE) and Gd-DTPA-bis(stearylamide) (Gd-DTPA-DSA), is increased 10-fold compared to bare silica NPs (Van Schooneveld et al., 2008). Lipid-coated silica NPs are also reported as lubricating drug nanocarriers against Osteoarthritis (OA) disease (Sun et al., 2018) or carrying bovine hemoglobin toward an as a erythrocyte mimic system (Tu et al., 2018). In both cases, experimental results showed that the silica NPs coated with SLBs, respectively, composed by DSPC and DOPC/DOPE/PEG_2000_DOPE greatly reduced the friction coefficient in comparison with bare NPs. However, the rapid clearance by the immune or excretory system can limit the efficacy of this kind of NPs. These limitations can be overcome by introducing PEG ligands or peptides which allows for cell targeting in the SLB on silica NPS. A large variety of lipids or membrane bound components can be included in the SLB. As an example, cholesterol is included to limit the fluidity of the SLB and avoid leakage issues related to fluid lipid coating. The use of PEG lipids has been suggested to improve the nanoparticle colloidal stability. PEG chains can also provide sites for chemical conjugation with small molecules acting as targeting agents, typically peptides or antibodies, and improve the NP circulation time (Ashley et al., 2011; Butler et al., 2016).

Generally, AuNPs are used for cancer diagnostics, photothermal therapy or as biosensor, thanks to their high extinction coefficient and the high photostability (higher than the commonly used organic fluorophore). These properties enable the use of the electromagnetic radiation to detect the AuNPs as well as to reveal the interactions with biological molecules. A lipid-coating composed by a SLB of dimethyldioctadecylammonium bromide (DODAB)/di-oleoyl-phosphatidylethanolamine (DOPE) can be used to improve NP biocompatibility by preventing non-specific binding of biomolecules on the NP surface and, at the same time, to host active molecules such as drugs or cell-targeting agents, such as folic acid and DNA (Du et al., 2015, 2017). The lipid-coating on NPs can be suitably designed for controlled release of active molecules embedded among the lipids. DPPC molecules were used for AgNPs and AuNPs thermo-responsive coating (Leo et al., 2013). DPPC is a thermoresposive phospholipid, which has a transition temperature from the gel to the liquid phase at 41°C. DPPC phase transition can be used to trigger the release of active molecules: DPPC in the gel-phase forms a particularly rigid bilayer, while the transition to the more-fluid liquid phase makes the DPPC coating prone to release the molecular cargo (Kang and Ko, 2015; Hamilton, 2017). Lipid-coated AgNPs can be also used as *in vitro* biosensors to study the molecular structure of soluble proteins which binds the lipid coating on the NP surface (Bhowmik et al., 2015).

**Figure 7:**
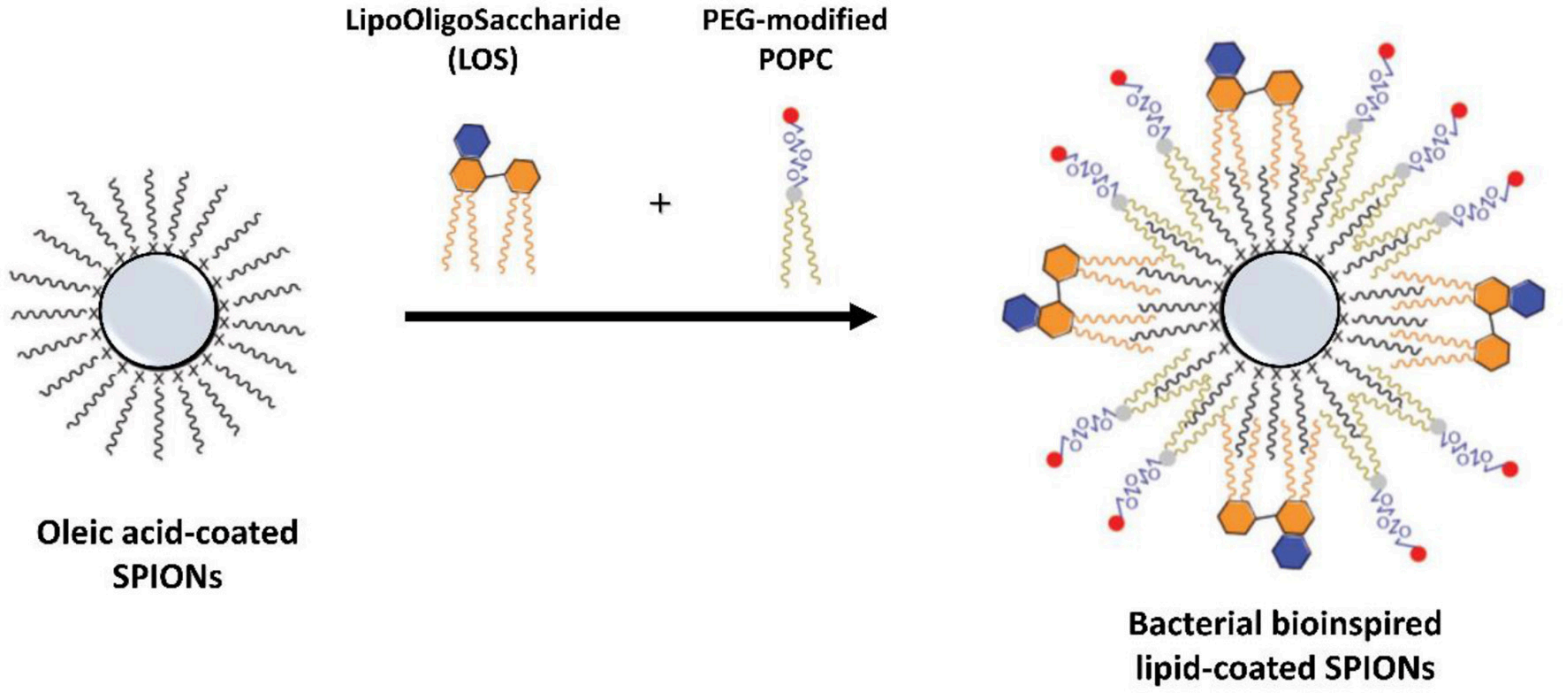
Illustration of SPIONs coated with a HLB containing a bacterial lipooligosaccharide (LOS) in the external leaflet as bioinspired nanostructures for cancer immunotherapy [adapted with permission from (Traini et al., 2019). Copyright (2019) WILEY-VCH].

SPIONs with a lysophosphocholine (LPC) coating were recently tested for applications as MRI contrast agents by *in vitro* and *in vivo* studies as well as carrier of an amphiphilic drug (Luchini et al., 2015, 2016b). These experimental tests prove that SPIONs coated with a HLB, with 18LPC molecules in the outer layer, are performing contrast agents, with favorable long circulation and enhanced MRI signal (T_2_). The versatility of these lipid-coated SPIONs was also improved by the successful introduction of the anticancer ruthenium complex ToThy-CholRu (Simeone et al., 2012) within the LPC coating. As demonstrated by *in vitro* bio-screens performed on Wistar rats and MCF-7 cells, respectively, these 18LPC-ToThyCholRu-coated SPIONs show high potential for the simultaneous detection and therapy of cancer (Luchini et al., 2015, 2016b). Similarly, multifunctional SPIONs coated with a SLB of lipoid phosphatidylcholine (PC) and cholesterol, also conjugated with a near-infrared fluorescence agent and further functionalized with a tumoral hepatocytes-targeting polymer, showed an high stability in physiological conditions; in addition, they demonstrated prominent tumor-contrasted imaging performance both on fluorescent and T_2_-weighted magnetic resonance (MR) imaging modalities in a living body (Liang et al., 2017). More recently, SPIONs with a HLB composed by a PEG-modified POPC and a lipooligosaccharide (LOS) derived from the plant pathogen as TLR4 agonists, were designed and developed as a cancer vaccine platform (Traini et al., 2019). This approach allows to produce stable bacteria-mimicking nanostructures for the development of cancer immunotherapies (Figure 7), proposing a novel bioinspired kind of multifunctional lipid-coated NPs with improved biomedical properties. Also, thermo-responsive SPIONs coated with DPPC and DPPG phospholipids were used to implement the controlled release of the anticancer camptothecin (CPT) drug (Allam et al., 2013).

Another interesting biomedical field where lipid-coated SPIONs can be used is to generate/scavenge Reactive Oxygen Species (ROS) thanks to the presence of semiconductor oxide inorganic core which can be used to treat cancer cells. Recently, lipid-coated magnetic mesoporous silica NPs doped with cerium oxide (CeO_2_) and iron oxide (Fe_3_O_4_) nanoparticles were recently designed (Cha et al., 2018). This kind of NPs shows both potent anti-inflammatory therapeutic effects *via* scavenging ROS radicals and an increasing efficacy of MRI enhancement in the perihematomal area, also representing the first nanobiomaterial that successfully showed theragnostic effects in the intracerebral hemorrhage (ICH), caused by the sudden rupture of an artery within the brain. QDs coated with a SLB of PEG-phospholipids were used as relevant probes for NIR (near infrared) whole-body imaging. Experimental evidences demonstrate that these functionalized QDs tend to rapidly accumulate in the tumor and produce a highly localized fluorescence signal that is suitable for tumor imaging and quantification (Papagiannaros et al., 2009).

## Perspectives on the Future Implementation of Lipid-Coated Nanoparticles

The number of synthetic strategies proposed to produce nanomaterials for biomedical applications is constantly growing. New suitable materials which can be brought to the nanometer scale allows to increase the library of potential nanodevices for various applications. Specifically, a great attention is currently dedicated to the development of multifunctional, bio-compatible and selective nanomaterials. In this context, lipid-coated NPs arouse great interest thanks to their capability to combine the biocompatibility of the lipid coating with the physical properties of the inorganic core. Indeed, the lipid coating improves or induces a significant NP colloidal stability in aqueous media, allowing the achievement of stable medical formulations, and can host active molecules, such as drugs, cell targeting agents, or contrast agents for multimodal detection and treatment of severe pathologies, such as cancer. Consequently, the lipid-coating appears as a powerful strategy to design and produce multifunctional nanocarriers which can strengthen the advance of the personalized medicine.

In this review, we highlighted the currently developed strategies for the synthesis and production of lipid-coatings on inorganic NPs. Particularly, a specific attention was focused on the preparation methods as function of the nature of lipids and of the inorganic cores (i.e., silica, gold, silver, iron oxide, quantum dots) which are widely studied and proposed in nanomedicine (Table 1). Actually, different synthetic protocols have been developed to obtain multifunctional biocompatible and bioinspired NPs with controlled sizes and modulated surface properties. At the same time, a great reproducibility in the design and formulation of lipid-coated NPs makes them promising candidates for biomedical applications. Indeed, all the selected nanosystems described in this review show interesting and promising properties, as demonstrated by the *in vitro* and *in vivo* experiments. Further development of this approach for nanomedicine can be achieved by extending the range of inorganic NPs, in terms of their chemical nature (i.e., semiconductor oxides instead of metals or hybrid composition), surface and structural properties (i.e., hydrophobic/hydrophilic grade, bulk/surface defects, metal doping), morphology. On the bases of these peculiar features, the application of these nanomaterials could be exploited to a greater number of pathologies. Finally, the possibility to insert/conjugate other bioactive molecules (i.e., lipopolysaccharides, aptamers) to the lipid coating could extend the NPs multifunctionality.

Nevertheless, many current studies on nano-biointerfaces are single-parametric, rendering the conclusions drawn less predictive in other scenarios. Different and combined physicochemical parameters can exert significant effects on the NP fates and biological performance upon administration. For this reason, a broader knowledge of property-structure-functionality relationships appears actually pivotal for a further application of these nanomaterials. Indeed, the physico-chemical interactions of NPs and biological systems (e.g., different cell components) can induce biological responses at multiple levels, ranging from biomolecular (e.g., protein absorption, complex disassembly, enzyme inhibition) and subcellular level (e.g., membrane disruption, functional loss of organelles) to cellular (e.g., division, differentiation, migration, death) and tissue level responses (e.g., inflammation, fibrosis, carcinogenesis). Moreover, a greater knowledge must be acquired about the efficiency of these NPs as carriers through fine capillary blood vessels and/or lymphatic endothelium, as well as the circulation duration and their accumulation in target tissues. These highlight the need for a further enhancement in the design of lipid-coated NPs, presenting a greater specificity toward the target cells depending on the disease to treat, a wider mimesis with respect to the immune system, as well as an implemented improved dynamic flow behavior.

In this scenario where chemistry, physics and biology are strikingly interconnected, it is evident the need for the development of suitable tools to fully understand the potential impact of nanoparticle-based system during their actual use. This should be the central and yet challenging aim for the future studies to bring systems such as lipid-coated NPs from the lab to their effective use in daily medicine.

## Author Contributions

All authors listed have made a substantial, direct and intellectual contribution to the work, and approved it for publication.

### Conflict of Interest Statement

The authors declare that the research was conducted in the absence of any commercial or financial relationships that could be construed as a potential conflict of interest.
